# Screening methods for thermotolerance in pollen

**DOI:** 10.1093/aob/mcae067

**Published:** 2024-05-07

**Authors:** Madeleine Stokes, Anja Geitmann

**Affiliations:** Department of Plant Science, Faculty of Agricultural and Environmental Sciences, McGill University, Montreal, Canada; Department of Plant Science, Faculty of Agricultural and Environmental Sciences, McGill University, Montreal, Canada

**Keywords:** Fertilization, heat stress, pollen, pollen viability, pollen vigour, plant reproduction, pollen development, pollen tube, pollen morphology, thermosensitivity, thermotolerance

## Abstract

Plant reproduction is highly susceptible to temperature stress. The development of the male gametophyte in particular represents a critical element in the reproductive cycle with high sensitivity to elevated temperatures. Various methods have been used to test the effect of temperature stress on pollen performance or to determine the degree of susceptibility of given species and genotypes. The information gained informs the development of new crop varieties suited to grow under warmer conditions arising through climate change and facilitates predicting the behaviour of natural populations under these conditions. The characterization of pollen performance typically employs the terms ‘pollen viability’ and ‘pollen vigour’, which, however, are not necessarily used consistently across studies. Pollen viability is a nominal parameter and is often assayed relying on cellular features as proxy to infer the capability of pollen grains to germinate and complete double fertilization. Alternatively, pollen germination can be determined through *in vitro* growth assays, or by monitoring the ability of pollen tubes to complete different progamic steps *in vivo* (ability to reach an ovule, release sperm cells, lead to seed set). Pollen vigour is an ordinal parameter that describes pollen tube growth rate or the efficiency of pollen tube growth as inferred by its morphology or growth pattern. To ensure consistent and relevant terminology, this review defines these terms and summarizes the methodologies used to assess them.

## INTRODUCTION

Temperature is an important influence on ecosystems and affects them in various ways. An increase in average temperature might facilitate the expansion of a species’ habitat into higher latitudes or higher elevations as temperatures become more suited to its survival in those regions. Inversely, increasing temperatures may push species from certain regions when the conditions start compromising plant survival or ability to reproduce. The climate to which a species is adapted must be compatible with key stages of their annual life cycle, such as blooming and reproduction. Pollen development and fertilization are weak links in the plant reproductive cycle and dramatically influence fruit development and seed set. Pollen exposed to abiotic stress resulting in sterility has been identified as an agricultural problem impacting productivity of multiple crops, including cereals and legumes (Powell *et al.*, 2012; Chaturvedi *et al.*, 2021). As a consequence of climate change, the frequency and intensity of heat waves are predicted to increase, compounded by an increase in average global temperatures. The duration of heat stress and the magnitude of the temperature increase during the stress impact the severity of the effect, with longer times and higher temperatures typically resulting in increased levels of damage (Colombo and Timmer, 1992). The combination of fragmented landscapes and rapid climate change has the potential to overwhelm the capacity of plant populations to adapt to changing conditions and consequently alter their genetic composition (Jump and Peñuelas, 2005). Gene flow by pollen can significantly affect the genetic structure within and among plant populations by means and distance of dispersal. When considering the effect of climate change on pollination-mediated gene flow, both pollen viability and vigour impact the probability that a pollen grain deposited on a stigma leads to the generation of a viable seed. Elevated overall temperatures and short-term temperature events have the potential to negatively impact fruit development and seed set, ultimately posing a threat to crop yield and thus food security.

**Fig. 1.**
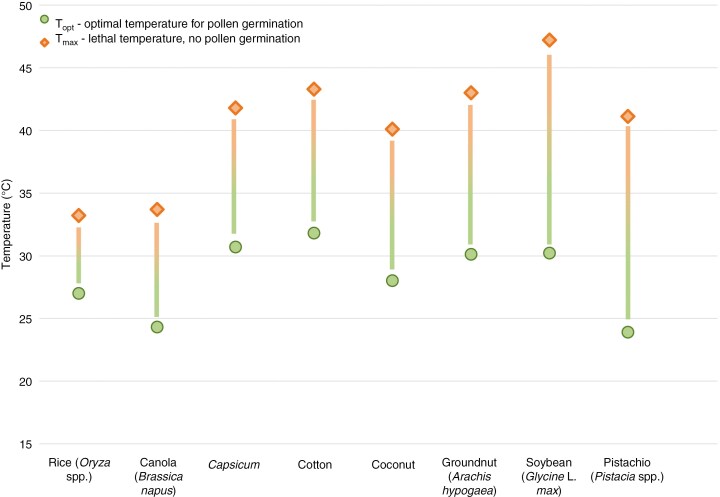
Optimal temperature vs lethal temperature stress for pollen germination (T_opt_ vs T_max_ based on mean cardinal temperatures) across different species, including rice (Coast *et al.*, 2016), canola (Singh *et al.*, 2008), capsicum (Reddy and Kakani, 2007), cotton (Kakani *et al.*, 2005), coconut (Ranasinghe *et al.*, 2018), groundnut (Kakani *et al.*, 2002), soybean (Salem *et al.*, 2007), and pistachio (Acar and Kakani, 2010).

Heat stress can induce changes in cell division processes during microsporogenesis, as well as the transcriptome, proteome, and/or the metabolome of the developing pollen (Kaplan *et al.*, 2004; Hedhly, 2011; Lohani *et al.*, 2020). Environmental stress on male gametogenesis impacts various biological processes, affecting important cytological mechanisms, such as cytoskeletal dynamics, tapetal endoplasmic reticulum stability, sugar metabolism and oxidative stress (De Storme and Geelen, 2014). Pollen sterility resulting from heat stress experienced during microgametogenesis in the anther can be caused indirectly as a result of the degradation of the tapetum or by a direct effect on the male gametophyte proper (Malik and Zhao, 2022). During the progamic phase, high temperature stress impairs pollen germination, alters pollen tube formation and growth, and can lead to poor pollen competition and selection (Raja *et al.*, 2019). The difference between optimal temperature (T_opt_) and lethal temperature stress (T_max_, 0% pollen germination rate) for pollen germination was found to be as small as 7 °C (Fig. 1). Investigating various plant species and cultivars for the susceptibility of their reproductive processes to temperature stress is therefore important and requires a consistent, reproducible and rigorous approach to measuring and characterizing these traits. Testing the functional quality of pollen allows predicting the fertility of parent plants and hybrids in genetic experiments and plant breeding and examining the state of pollen after storage, and it is essential for gaining mechanistic insight into the processes governing pollen–stigma interaction, incompatibility systems and fertilization. Here we discuss the common terminology used to describe pollen fitness and the corresponding methods of testing. We focus in particular on methods used in the context of temperature stress. We aim to define and standardize the terminology to enable replicable and reproducible research that facilitates application for breeders, agronomists, plant biologists, and geneticists.

## MALE GAMETOPHYTE DEVELOPMENT IS SENSITIVE TO HIGH TEMPERATURES

**Fig. 2.**
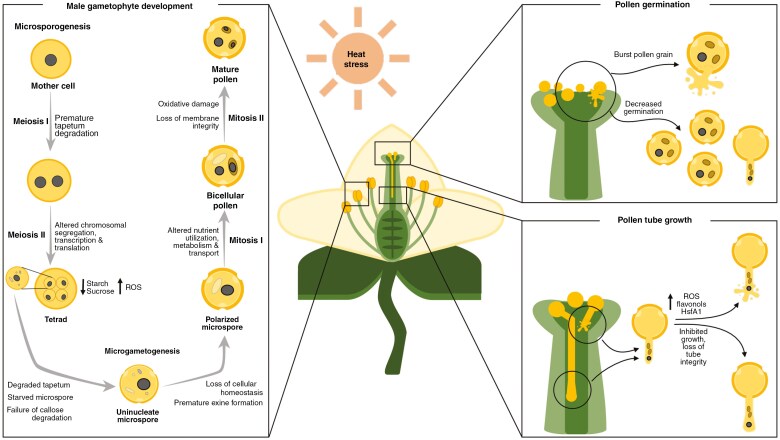
Stages during male gametophyte development susceptible to heat stress. (1) During microsporogenesis, meiosis I and II can be affected by early breakdown of the tapetum cell layer, impacting nutritional supply to the developing pollen, heat-induced defects in meiosis by increasing the frequency of chromosomal crossing-over and homologous recombination, decrease in sucrose/starch reserves while ROS levels increase, impacting the ability of the microspores to be released; during microgametogenesis, the anther can exhibit a degraded tapetum, the microspore can exhibit failure of callose degradation, premature pollen exine formation, loss of cellular homeostasis, and mitosis I and II can be affected by altered sugar-starch utilization, metabolism and transport, oxidative damage and loss of cell membrane integrity, which can ultimately lead to male sterility. (2) Heat-induced decrease in pollen germination rate, pollen bursting or showing morphological abnormalities (shrivelling or altered pollen grain size). (3) Pollen tube growth can be slowed and inhibited, exhibiting changes in morphology (swelling, bursting).

In angiosperm sexual reproduction, three phases can be distinguished: male and female gametophyte formation, the progamic phase and embryo development. While all processes of plant reproduction are susceptible to heat stress, pollen is particularly sensitive to elevated temperatures through all phases of male gametophyte development, while the formation of the embryo sac (female gametophyte) is more robust (Hedhly, 2011). Heat stress can negatively impact all phases of male gametophyte development and fertilization (Fig. 2). The change of morpho-anatomical structures in male reproductive tissues under heat stress can impact various pollination-related events, including pollen contact with the stigma, adhesion and hydration, and subsequent pollen germination, tube growth and fertilization of the ovule (Santiago and Sharkey, 2019).

Male gametophyte development is more sensitive at the early stages (anther wall development, microsporogenesis, microgametogenesis) than later stages (pollen maturation, anther dehiscence) or during the progamic phase (pollen hydration, germination, pollen tube growth) (Raja *et al.*, 2019). High temperature stress has both short- and long-term implications for the events occurring during microgametogenesis, including meiosis, mitosis and exine development (Garcia *et al.*, 2017). In barley plants (*Hordeum vulgare*), heat stress (30 °C day/25 °C night) administered during anther development was found to affect the sporogenous tissue directly and to interfere with the surrounding tissues impacting nutrient supply to the maturing pollen (Oshino *et al.*, 2007). Anther development occurring at the upper limit of the viability temperature range for a given species, usually between 30 and 35 °C (Branch and Sage, 2018), negatively impacts meiosis by inducing the abortion of uninucleate microspores (Sage *et al.*, 2015). Direct effects on pollen development include interference with meiosis through modulating chromosome crossover and homologous recombination (Boyko *et al.*, 2005; Francis *et al.*, 2007) or preventing chromosome separation entirely leading to unreduced diploid male gametes (Pecrix *et al.*, 2011; De Storme and Geelen, 2020). Chickpea plants gradually exposed to heat stress over 12 d (temperature increased daily by 1 °C, e.g. 28 to 40 °C during the day and 16 to 25 °C during night) during early flowering produced anthers with increased numbers of locules, reflecting changes in anther development phase 1 (anther cell differentiation) and anther development phase 2 (pollen grain differentiation) (Goldberg *et al.*, 1993), and pollen with reduced viability (Devasirvatham *et al.*, 2012). A reduction in pollen viability due to high temperature stress has been correlated to changes in the level and composition of several groups of metabolites that play important roles in pollen development by providing sufficient nutrients and protecting against abiotic stress (Paupière *et al.*, 2014). Heat stress can disrupt the metabolic equilibrium by altering the composition of carbohydrates, proteins, lipids, phytohormones, flavonoids and alkaloids, ultimately affecting pollen germination and pollen tube growth (Pressman *et al.*, 2002; Sato *et al.*, 2002; Goel *et al.*, 2023).

## Pollen tube growth is affected by heat

Pollen tube growth corresponds to the progamic phase, which in some species can be completed within just a few hours. It is the most rapid cellular growth process in the plant kingdom and relies on proficient exocytosis of cell wall material and cell–cell communication to allow the tube to rapidly elongate through the pistillar tissues towards an ovule to deposit two sperm cells. At the molecular level, heat stress affects cell wall composition of pollen tubes likely resulting from interference with cytoskeletal functioning and vesicle transport, which affect the delivery of cellulose and callose synthases to the cell surface (Parrotta *et al.*, 2016). High temperature stress also increases levels of signalling molecules such as ROS, inhibiting pollen tube growth and causing them to swell and burst (Muhlemann *et al.*, 2018). Damage by high levels of ROS is mitigated by feedback mechanisms including flavonols or peroxidases, which scavenge ROS (Zhang *et al.*, 2018). ROS accumulation under heat stress induces the expression of *HEAT SHOCK TRANSCRIPTION FACTOR A1* (*HsfA1*), which generates a heat shock response via gene expression (Yoshida *et al.*, 2011). The transcription profile of maturing tomato (*Solanum lycopersicum*) pollen exposed to short-term heat stress (43–45 °C for 2 h) revealed the accumulation of the small heat shock protein (*HSP*) gene family along with important regulators of heat stress, such as ROS scavengers, sugars, plant hormones and regulatory genes (Frank *et al.*, 2009). Depending on the severity and duration of high temperature stress, a heat stress response can involve an unfolded protein response in the endoplasmic reticulum (Mittler *et al.*, 2012; Bokszczanin and Fragkostefanakis, 2013). While the heat stress response at non-lethal temperatures is able to restore cellular homeostasis, excessive and/or long-term elevated temperatures have the potential to activate programmed cell death.

## QUANTIFYING POLLEN PERFORMANCE

Quantifying pollen performance can serve to determine the effect of a stress applied during pollen development in the anther or to establish the susceptibility of the male gametophyte to stress applied during the progamic phase. The latter obviously requires experiments involving pollen germination whereas the former can be done either by assessing the behaviour of the germinated pollen or by inferring pollen performance from cellular parameters observed in the ungerminated pollen grain and serving as proxy.

Pollen performance can be scored based on different aspects of pollen fitness (Table 1). The term ‘pollen viability’ has been used in a variety of ways and, for consistency, we propose using it for criteria involving *nominal* scoring (yes/no) of the performance determined at the level of the individual pollen grain (e.g. ability to germinate, deliver sperm cells, or result in seed set). The cell population-based outcome is presented as a ratio. ‘Pollen vigour’ typically measures time as the dependent variable and, for consistency, we propose using it for *ordinal* scores, e.g. by recording the time it takes for pollen to germinate or grow a pollen tube to a certain length or, inversely, recording pollen tube length after a defined time. The population-based outcome is typically presented as an averaged value. Pollen tube morphology falls under pollen vigour, being indicative of an ordinal property as it relates to the speed in reaching and efficiency in targeting an ovule or completing double fertilization.

**Table 1.** Methods of testing pollen viability and pollen vigour (* denotes papers studying pollen in relation to high temperature stress). Note that some studies use *in vitro* pollen germination as a method of testing pollen viability while other studies use *in vitro* germination to correlate a given pollen viability staining method to determine its accuracy.

Parameter	Definition	Method of measuring	Pollen Parameters Measured	References
Pollen viability	Capacity of pollen to live, grow, germinate or develop (Dafni and Firmage, 2000).	Pollen grain morphology	Cell wall ultrastructure, exine ornamentation, cytoplasmic volume	Koti *et al.*, 2005*; Bhattacharya and Datta, 2011; Jiang *et al.*, 2015*; Hinojosa *et al.*, 2019*
		Impedance flow cytometry (ICF)	Plasma membrane integrity	Heidmann *et al.*, 2016; Heidmann and Di Berardino, 2017; Luria *et al.*, 2019*; Ascari *et al.*, 2020*a*; Rafiq *et al.*, 2021
		FDA (FCR) test	Plasma membrane integrity, enzyme activity	Dupuis and Dumas, 1990*; Shivanna *et al.*, 1991a*, b*; Trognitz, 1991; Sukhvibul and Considine, 1993; Khatun and Flowers, 1995; Kelen and Demirtas, 2003; Vizintin and Bohanec, 2004; Nepi *et al.*, 2005; Abdelgadir *et al.*, 2012; Dutta *et al.*, 2013; Mazzeo *et al.*, 2014; Muhlemann *et al.*, 2018*; Ascari *et al.*, 2020*b*; Impe *et al.*, 2020; Al-Najm *et al.*, 2021
		Iodine potassium iodide (I-KI; Lugol’s stain; Baker’s procedure)	Presence of starch	Baker and Baker, 1979; Rodriguez-Riano and Dafni, 2000; Huang *et al.*, 2004; Bhattacharya and Datta, 2011; Mandal *et al.*, 2011; Abdelgadir *et al.*, 2012; Djanaguiraman *et al.*, 2013*; Melloni *et al.*, 2013; Mukherjee and Datta, 2013; Soares *et al.*, 2013; Sulusoglu and Cavusoglu, 2014; Luo *et al.*, 2020; Al-Najm *et al.*, 2021
		Triphenyl tetrazolium chloride (TTC)	Cellular respiration	Norton, 1966; Trognitz, 1991; Khatun and Flowers, 1995; Kelen and Demirtas, 2003; Huang *et al.*, 2004; Bhattacharya and Datta, 2011; Abdelgadir *et al.*, 2012; Gaaliche *et al.*, 2013; Soares *et al.*, 2013; Sulusoglu and Cavusoglu, 2014; Hinojosa *et al.*, 2019*; Luo *et al.*, 2020; Al-Najm *et al.*, 2021; Buchner *et al.*, 2022
		MTT (thiazolyl blue tetrazolium bromide)	Presence of dehydrogenase enzymes	Khatun and Flowers, 1995; Rodriguez-Riano and Dafni, 2000; Vizintin and Bohanec, 2004; Douglas and Freyre, 2010; Abdelgadir *et al.*, 2012; Watts *et al.*, 2013; Luo *et al.*, 2020; Li *et al.*, 2023
		Alexander’s stain	Presence of cytoplasm	Alexander, 1969; Atlagic *et al.*, 2012; Devasirvatham *et al.*, 2012*; Migdalek *et al.*, 2014; Tello *et al.*, 2018; Impe *et al.*, 2020; Al-Najm *et al.*, 2021; Buchner *et al.*, 2022;
		Acetocarmine	Presence of cytoplasm	Atlagic *et al.*, 2012; Devasirvatham *et al.*, 2012*; Malayeri *et al.*, 2012; Dutta *et al.*, 2013; Gaaliche *et al.*, 2013; Mazzeo *et al.*, 2014; Migdalek *et al.*, 2014; Luo *et al.*, 2020; Al-Najm *et al.*, 2021; Buchner *et al.*, 2022; Li *et al.*, 2023
		Aniline blue–lactophenol	Presence of cytoplasm	Khatun and Flowers, 1995; Vizintin and Bohanec, 2004; Mandal *et al.*, 2011; Abdelgadir *et al.*, 2012; Melloni *et al.*, 2013; Alexander, 2019; Al-Najm *et al.*, 2021; Masthigowda *et al.*, 2022*
		Benzidine–H_2_O_2_	Peroxidase activity	Chen *et al.*, 2013; Luo *et al.*, 2020; Li *et al.*, 2023
		Amido black	Presence of protein in cell wall	Bhattacharya and Datta, 2011; Mandal *et al.*, 2011; Mukherjee and Datta, 2013
		X-gal test	β-Galactosidase activity	Trognitz, 1991; Rodriguez-Riano and Dafni, 2000; Bhattacharya and Datta, 2011; Mandal *et al.*, 2011
		Isatin	Presence of proline	Firmage and Dafni, 2000
		*p*-Phenylenediamine	Presence of myeloperoxidase	Rodriguez-Riano and Dafni, 2000; Parzies *et al.*, 2005
		*In vitro* germination	Pollen germination rate (%) Pollen tube length (µm)	Matlob and Kelly, 1973*; Trognitz, 1991; Sukhvibul and Considine, 1993; Rodriguez-Riano and Dafni, 2000; Vara Prasad *et al.*, 2003*; Salem *et al.*, 2007*; Kafizadeh *et al.*, 2008*; Devasirvatham *et al.*, 2012*; Chen *et al.*, 2013; Dutta *et al.*, 2013; Mazzeo *et al.*, 2014; Jiang *et al.*, 2015*; Song *et al.*, 2015*; Alexander, 2019; Impe *et al.*, 2020; Buchner *et al.*, 2022
		Pollen tube integrity	Intact vs burst pollen tube	Muhlemann *et al.*, 2018*
	Ability of pollen to fertilize and lead to seed set (Shivanna *et al.* 1991a)	Semi-*in vivo* germination	Pollen tube ability to (a) navigate to target, (b) reach ovule, (c) deposit sperm cells	Zhong *et al.*, 2020
		In vivo germination	Ability to release sperm cells and cause seed set	Shivanna *et al.*, 1991a
Pollen vigour	Time taken for germination and time necessary for the tube to reach the embryo sac (Shivanna *et al.*, 1991*a*). Growth rate (unit of time): used to describe how conditions prior to/during pollen development or the progamic phase impact pollen germination and tube growth.	*In vitro*, semi-*in vitro* germination assays	Time for pollen to germinate (min) Pollen tube growth rate (µm/min) Pollen tube length (µm) achieved in a given time period	Shivanna *et al.*, 1991a*, b*; Rosell *et al.*, 2006; Karapanos *et al.*, 2010*; Song *et al.*, 2015*; Jiang *et al.*, 2015*
		Pollen tube morphology	Uniformity of pollen tube diameter Cell wall composition Pollen tube growth directional growth	Parrotta *et al.*, 2016*; Muhlemann *et al.*, 2018*; Çetinbaş-Genç, 2020*

**Fig. 3.**
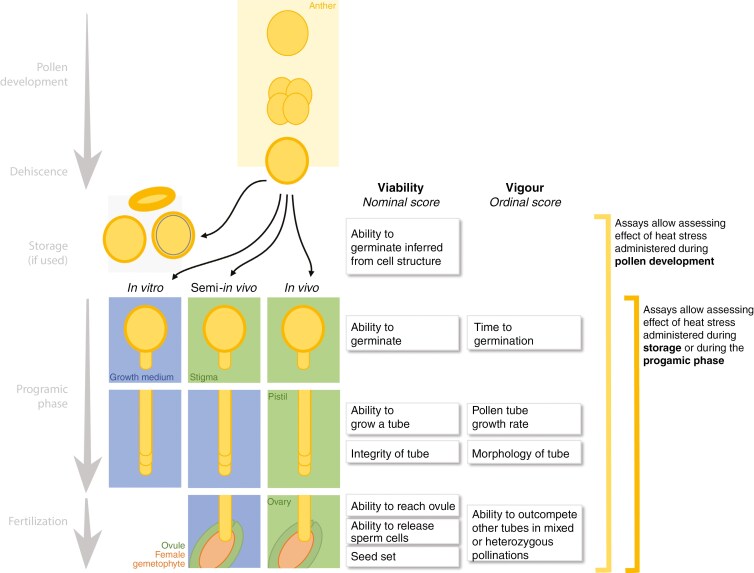
Schematic of different pollen performance assays based on different measuring parameters.

The terms ‘pollen viability’ and ‘pollen vigour’ therefore refer to different aspects of the progamic phase that can be used to assess pollen fitness in general and the effect of heat treatments specifically (Fig. 3). Pollen viability and vigour can be measured by a variety of experimental approaches and well-defined and reproducible methods of testing susceptibility to temperature stress are essential to ensure comparability of data and applicability for breeding. Many of these methods are also employed to study the molecular mechanisms underlying temperature stress response in pollen thus supporting breeding programmes to adapt to conditions caused by climate change.

### Pollen viability

Pollen viability has been defined as ‘having the capacity to live, grow, germinate or develop’ (Lincoln *et al.*, 1982) and ‘the ability of the pollen grains to perform its function of delivering sperm cells to the embryo sac following compatible pollination’ (Shivanna *et al.*, 1991*a*). There exist many methods to test pollen viability, and none is recognized to be universal. Rather than verifying whether the pollen actually germinates, pollen viability is often scored on ungerminated pollen, relying on cellular characteristics as a proxy. These characteristics include the external morphology and internal cellular and metabolic features. Different dyes are used for the latter, including the fluorochromatic reaction (FCR test), iodine potassium iodide (I-KI), triphenyl tetrazolium chloride (TTC), thiazole blue (MTT), Alexander’s stain, acetocarmine, lactophenol blue, and benzidine–H_2_O_2_. The FCR test uses fluorescein diacetate (FDA) to detect intact plasma membrane status and the presence of esterase activity, while I-KI detects the presence of starch. TTC detects cellular respiration and MTT detects the presence of dehydrogenase enzymes in the pollen grain; both are based on using tetrazolium salts. The presence of cytoplasm in pollen grains can be detected by Alexander’s stain, acetocarmine and aniline cotton blue. Other staining methods less cited, including amido black, the X-gal test, isatin and *p*-phenylenediamine, have been used to examine pollen viability based on the presence of protein in the cell wall, detection of β-galactosidase activity, detection of proline, and the presence of myeloperoxidase, respectively. From the scores established based on these proxy features, the ratio of viability within a pollen population is inferred. Scoring pollen grain viability in this manner is commonly used to assess the effect of heat stress applied during pollen development in the anther. When used on mature, ungerminated pollen, proxy features tend to generate results close to *in vitro* germination assays but in immature pollen or pollen with thick exine, stain-based tests have been found to under or overestimate viability (Luo *et al.*, 2020). Consequently, while staining methods are useful and quick, the result is often only a rough estimate of the pollen’s capability to germinate. The advantage of stain-based tests is their simplicity and the potential for automated scoring using e.g. a computer image analysis system that is trained to differentiate viable vs non-viable pollen grains based on stain hue and/or intensity. Another method to examine pollen viability is through impedance flow cytometry (IFC), which uses an electrical field to differentiate between viable and non-viable pollen grains based on differences in membrane integrity that are reflected in dielectric properties.

Alternatively, pollen viability can be evaluated based on the actual ability of the pollen to germinate, form a tube and/or lead to seed set. This can be done through *in vitro* and semi-*in vivo* germination assays in which pollen germination rate (%) and/or pollen tube length are scored, or the test can be conducted *in vivo* by scoring developing seeds (El Ahmadi and Stevens, 1979). Tests involving pollen germination can be used to assess the effect of heat stress administered during pollen development in the anther, and, unlike the stain-based tests, they can also be used to test the effect of heat stress applied during the progamic phase. In the following, the individual types of tests are elaborated in detail.

#### Pollen grain morphology.

To a certain degree, pollen viability can be inferred from the morphology of the pollen grains. For rough counting of ‘normal’ vs ‘aberrant’ grain morphologies, bright-field microscopy can be used. In soybean, altered pollen tube morphology was recorded in pollen grains exposed to high temperature stress (38/30 °C day/night), which was described as flattened, shrivelled and collapsed pollen grains (Salem *et al.*, 2007). Morphological abnormalities were more frequent in soybean genotypes classified as ‘heat-sensitive’ and ‘heat-intermediate’ compared with ‘heat-tolerant’ genotypes. For more detailed investigations of cellular processes that might have led to the formation of aberrant pollen grains, scanning electron microscopy (SEM) has been used to visualize pollen grain surface, while transmission electron microscopy (TEM) is required to assess the thickness of the wall or the ultrastructure of the cytoplasm. Fourier transform infrared spectroscopy (FTIR) and FT-Raman spectroscopy can be employed to analyse pollen surface composition (Jiang *et al.*, 2015; Kendel and Zimmermann, 2020) such as lipids in the pollen coat which are vital for pollen hydration (Dickinson, 1993). All principal biochemical constituents of pollen, including proteins, lipids, carbohydrates, carotenoids and sporopollenin, have been identified and detected in the spectra, and can be quantified. A study investigating how high temperatures impact the pollen cell wall in field pea found that high temperature (36 °C) caused a significant increase in intine thickness (Jiang *et al.*, 2015). While the analysis of pollen morphology is useful when attempting to deduce which cellular processes during pollen development may have been affected by a heat stress administered during pollen development, it is less reliable as a predictor of the actual ability of the pollen to germinate.

#### Detection of membrane integrity and enzyme activity.

The FCR test is based on esterase activity and an intact plasma membrane that can be detected by fluorescence microscopy when pollen grains are exposed to FDA. Specifically in pollen, the FCR test assesses two properties: the integrity of the plasma membrane and the activity of non-specific esterases in the cytoplasm of the vegetative cell (Heslop-Harrison *et al.*, 1984; Pinillos and Cuevas, 2008). The FCR test was first conducted to test pollen viability by Heslop-Harrison and Heslop-Harrison, (1970) on the premise that integrity of the plasma membrane of the vegetative cell is closely correlated with viability. When FDA is applied to pollen, non-fluorescent fatty acids esters of fluorescein freely enter the vegetative cell, where they may promptly be hydrolysed by esterases to release fluorescein, a polar and fluorescent molecule. If the vegetative plasma membrane is intact, fluorescein temporarily accumulates inside the pollen and the pollen emits fluorescence when exposed to excitation light (Shivanna and Heslop-Harrison, 1981). If the plasma membrane of the vegetative cell is damaged, the pollen grain does not produce fluorescence as fluorescein is unable to enter and accumulate in the cell where the enzymes responsible for hydrolysis are found. Pinillos and Cuevas, (2008) tested various parameters of the FCR test to optimize procedures that were not specified by Heslop-Harrison and Heslop-Harrison, (1970). Depending on the species, FDA concentration and incubation period need to be optimized, and the limited lifetime of the solution needs to be considered. Other work has been done to optimize protocols for the FCR test to enhance sample preparation and staining in *Juniperus communis* (Nepi *et al.*, 2005). A modified protocol combines the FCR test with propidium iodide (PI) to ensure that both viable and non-viable pollen grains can be detected using fluorescence microscopy settings at different emission wavelengths (Muhlemann *et al.*, 2018). Non-viable pollen grains become permeable to PI, which accumulates inside the grain, whereas viable pollen does not show cytoplasmic label with PI. Using this combined label on ten pollen taxa, pollen viability was estimated and used to create an automated assessment method of pollen viability using fluorescence signals (Ascari *et al.*, 2020*b*). The FCR test can be a quick and efficient way of estimating the percentage of viable pollen and has been shown to have a strong correlation with the rates of *in vitro* germination in many species, including tomato (Abdul-Baki, 1992), date palm (*Phoenix dactylifera*) (Al-Najm *et al.*, 2021), caprifig (*Ficus carica*) (Ilgin *et al.*, 2007), lily (Rhee *et al.*, 2003) and *Paeonia ostii* (Li *et al.*, 2023). Importantly, however, a high correlation between FCR and *in vitro* germination can only be expected when mature pollen is used, whereas immature pollen will result in an overestimation of germinability (Heslop-Harrison *et al.*, 1984). The reliability of the test is also species-dependent; in wheat (fresh pollen) (Impe *et al.*, 2020) and mango (cold-stored pollen) (Dutta *et al.*, 2013), the FCR test was found to overestimate pollen viability when compared with *in vitro* germination experiments. Nonetheless, the FCR test remains commonly used to test pollen viability.

#### Detection of starch content.

Mature pollen grains of most angiosperm species contain starch to provide energy reserves for germination, and its detection can be used as an indicator of pollen ability to germinate and perform fertilization. Submerging pollen grains in iodine dissolved in aqueous I-KI solution is a simple and versatile method to test for the presence of starch indicated by black or dark colour (Baker and Baker, 1979). In immature or poorly developed pollen, I-KI stain is typically absent or weak (Sulusoglu and Cavusoglu, 2014). In soybean, pollen viability after an exposure to heat stress for 14 days at the flowering stage (38/28 °C day/night) was tested using I-KI, and pollen round in shape and stained black was determined to be viable or living, in contrast to sterile pollen, which was observed to be misshaped/oval and stained yellow or light red (Djanaguiraman *et al.*, 2013). However, the detection of starch only allows confirming that pollen development had proceeded normally whereas it cannot detect whether the mature pollen might have been killed by a heat event once mature, during transfer or storage. Pollen viability in *Leymus chinensis* was assessed using I-KI, which failed to distinguish between fresh pollen and pollen killed by a 2h exposure to 80 °C: most of the heat-killed pollen treated with I-KI stained in the same manner as fresh pollen (Huang *et al.*, 2004). In the same study, *L*. *chinensis* was pollen-stained with TTC (see below for details), which was able to differentiate between viable pollen and heat-treated pollen.

#### Detection of cellular respiration.

Differentiation between metabolically active and inactive cells can be made based on an assessment of cellular respiration using TTC, a redox indicator (Norton, 1966). TTC is a colourless solution and in living cells it is reduced by oxidative and dehydrogenase enzymes to a dark red formazan complex (Smith, 1951). In cherry (*Prunus laurocerasus*) and grape (*Vitis vinifera* ), pollen viability has been determined using TTC by scoring pollen as viable, characterized by orange/bright red grains, and non-viable, observed as colourless or light pink pollen grains (Kelen and Demirtas, 2003; Sulusoglu and Cavusoglu, 2014). These results were confirmed by comparison with the FDA test, but the reliability of TTC as indicator for pollen viability varies significantly across species. TTC produced viability scores similar to *in vitro* germination experiments in pollen from pine (Cook and Stanley, 1960), maize (*Zea mays*) (Viéitez Cortizo, 1952), plum (Norton, 1966) and common ash (*Fraxinus excelsior*) (Buchner *et al.*, 2022), but overestimated viability in pollen from apple, grape, peach, pear, *Castanea mollissima* (Oberle and Watson, 1953) and *Castanea henryi* (Luo *et al.*, 2020).

#### Detection of dehydrogenase enzymes.

Thiazole blue (MTT) is a dye that can be used to determine the presence of dehydrogenase as an indication of pollen viability. Viable grains change colour from yellow to deep pink/purple or black. Tested across eight different species, MTT results showed a high correlation with *in vitro* pollen germination assays using fresh pollen (Rodriguez-Riano a Dafni, 2000). However, it was noted that MTT should be analysed with caution as it resulted in many different colour tonalities and some killed pollen was stained, albeit much lighter than the fresh pollen. MTT has been successfully used in *Caesalpinia crista* (Li *et al.*, 2004), *Iris* (Sapir *et al.*, 2005), *Caulokaempferia coenobialis* (Zingiberaceae) (Wang *et al.*, 2005) and *Oxalis debilis* (Luo *et al.*, 2006), but was found to be unreliable in two *Nolana* species (Douglas and Freyre, 2010) and *Jatropha curcas* (Euphorbiaceae) (Abdelgadir *et al.*, 2012).

#### Detection of cytoplasmic activity.

The presence of cytoplasm can be detected using bright-field optics with Alexander’s stain, which combines acid fuchsin and orange G to stain the cytoplasm red/pink with malachite green, which stains the cellulose of cell walls green (Alexander, 1969). This method assays pollen viability based on the assumption that pollen containing cytoplasm can be regarded as viable. Pollen grains red/pink in colour are determined to be viable, while in non-viable pollen grains (not containing a cytoplasm), the green-stained cell walls are prominent and red stain is absent. Processing tools, such as PollenCounter, have been developed to perform automatic counting of pollen grains based on the two-colour Alexander’s modified staining, speeding up the process of estimating pollen viability (Tello *et al.*, 2018). Alexander’s staining shows good correlation with *in vitro* pollen germination rates and is simple to conduct, making it appropriate for large-scale breeding programmes (Atlagic *et al.*, 2012). However, loss of viability during storage is not detected, producing false-positives (Heslop-Harrison *et al.*, 1984; Impe *et al.*, 2020). Alexander’s stain uses chloral hydrate, phenol and mercuric chloride, all of which are highly toxic chemicals that can be difficult to procure. A simplified approach of Alexander’s staining was developed eliminating use of some of the toxic chemicals and allowing wider application of this method (Peterson *et al.*, 2010).

An alternative dye-based method to label the pollen grain’s cytoplasm is acetocarmine. Red stain is interpreted to indicate a viable pollen grain whereas absence of stain indicates non-viability (Migdalek *et al.*, 2014). Acetocarmine has been used to test pollen viability in mango (*Mangifera indica*) (Dutta *et al.*, 2013), *Solidago* × *niederederi* (Migdalek *et al.*, 2014), caprifig (*Ficus carica*) (Gaaliche *et al.*, 2013), *Cercis siliquastrum*, *Medicago sativa*, *Robinia pseudoacacia*, *Melilotus officinalis*, *Trifolium repens* and *Sophora alopecuroides* (Malayeri *et al.*, 2012). However, when compared with *in vitro* pollen germination assays, acetocarmine often overestimates pollen viability (Dutta *et al.*, 2013).

Aniline (cotton) blue in lactophenol stains viable pollen grains with a cytoplasm blue and pollen grains without or low quantities of cytoplasm remain unstained (Hauser and Morrison, 1964). Lactophenol blue has been used to estimate pollen viability in sugarcane (Melloni *et al.*, 2013), *Jatropha curcas* (Abdelgadir *et al.*, 2012) and cucumber (Vizintin and Bohanec, 2004). Similar to other staining approaches, lactophenol only gives a rough estimation of viable pollen and may not reliably differentiate between viable and non-viable pollen in some species (Abdelgadir *et al.*, 2012).

#### Detection of peroxidase activity.

Benzidine–H_2_O_2_ is used to detect pollen viability based on the oxidation of benzidine by peroxidase in the presence of hydrogen peroxide. The reaction in the pollen grain is accompanied by bubbles, which derive from the emission of oxygen freed by the hydrogen peroxide by catalase (King, 1960). Viable grains of potato and tomato quickly enlarge and appear colourless and ovoid in shape, while non-viable grains become blue and do not enlarge. Benzidine–H_2_O_2_ has been used to distinguish viable pollen from non-viable pollen in *Castanea mollissima* and *Castanea henryi*, and *Paeonia ostii* (Luo *et al.*, 2020; Li *et al.*, 2023).

#### Other staining methods.

There exist many other staining methods for determining pollen viability, many of which are not widely used or well established, nor have they been employed in the context of heat stress studies, but we opt to mention some of them for completeness. They include the detection of protein in the cell wall of mature pollen by amido black (Mandal *et al.*, 2011), the detection of β-galactosidase activity by the X-gal test using 5-bromo-4-chloro-3-indolyle-β-galactoside (Rodriguez-Riano and Dafni, 2000), the detection of proline after incubation with isatin (Firmage and Dafni, 2000), and the evidence of myeloperoxidase in viable pollen grains using p-phenylenediamine (Rodriguez-Riano and Dafni, 2000).

#### Impedance flow cytometry.

Impedance flow cytometry has been used to estimate pollen viability and is based on the principle that, due to differences in the plasma membrane status, viable and non-viable cells have different electrical properties that can be distinguished by passing the cells through an electric field (Heidmann *et al.*, 2016). IFC is performed by pumping suspended cells through a highly sensitive microchip designed to generate an electric field. The passage of a cell through the electric field causes a characteristic electrical signal, which is measured by the cytometer as an impedance signature, allowing differentiation of viable cells from non-viable cells. IFC is advantageous as it is non-destructive and has been determined to be a quick, reliable and standardized method for estimating pollen viability in multiple species (Heidmann and Di Berardino, 2017). The principal drawback of IFC is the cost of the instrument and the need for calibration for each species. Initial challenges related to low reliability were related to the relatively large dimensions of the microfluidic channels compared with pollen size, causing current leakage. This problem has been addressed in more recent designs with narrower channels (Chen *et al.*, 2011), or by sandwiching the cell between two insulating fluid layers (e.g. insulating fluid flow) (Bernabini *et al.*, 2011). IFC has successfully determined viability in pollen from species including hazelnut (*Corylus avellana*) (Ascari *et al.*, 2020*a*), *Cannabis sativa* (Rafiq *et al.*, 2021), pepper (*Capsicum annuum*) (Lin *et al.*, 2022) and wheat (Impe *et al.*, 2020).

#### In vitro germination.

Most of the above-mentioned methods do not allow detecting if a pollen grain’s viability was affected at any time after completion of maturation, including during storage. Doing so requires assaying whether the grain actually germinates. Ideally, one would assess the germination behaviour *in vivo*, on a stigma, to provide the pollen with optimal conditions. However, quantifying germination percentage on the stigma is laborious if not impossible and hence typically the test is done *in vitro*. If an *in vivo* setup is desired, rather than determining germination ratios on the stigma, it is more feasible to quantify pollen tube growth, and often the length of the longest pollen tubes growing into the pistil at a given time is determined, or, of course, seed set.

*In vitro* pollen germination can assess the consequences of abiotic stress during pollen development similar to the above-mentioned staining methods, but, unlike these proxy methods, it can also assess the effect of heat exposure during storage and during the progamic phase. In general, a direct relationship exists between pollen viability and germination capability in many species, making *in vitro* germination a more direct indicator of pollen viability compared with the proxy methods (Stanley and Linskens, 1974). *In vitro* germination requires optimizing the growth conditions to simulate the conditions of the style–stigma through the use of germination media containing the nutrients required to initiate pollen germination and sustain pollen tube growth in a given species (Soares *et al.*, 2013). In addition to the chemical composition of the growth medium, germination behaviour is subject to quorum sensing, thus making the density of pollen grains in the tested sample volume critical (Heslop-Harrison *et al.*, 1984). Under high temperatures and relative humidity (38/45 °C, >95 % relative humidity) in tobacco pollen, pollen tube growth is considerably slower in *in vitro* assays when compared with *in vivo* germination experiments (Shivanna *et al.*, 1991*b*). This suggests that the suboptimal chemical conditions *in vitro* make pollen germination and tube growth more susceptible to temperature stress compared with *in vivo* germination conditions. In *Arabidopsis*, optimized *in vitro* conditions involve a stiffening agent such as agarose and the presence of a squashed pistil in the medium (Bou Daher *et al.*, 2009). Consideration of the limited ability of *in vitro* setups to mimic *in vivo* situations is important when translating results from *in vitro* pollen germination experiments to breeding programmes. The principal drawback of *in vitro* germination vs pollen grain staining methods is the substantial effort required to optimize the growth medium for each species and cultivar.

#### Pollen tube integrity.

Pollen tube integrity relates to the ability of pollen to deliver the sperm cells. The maintenance of pollen tube integrity is vital for pollen tube growth towards the ovules, and tube rupture should only take place once the pollen tube reaches the female gametophyte inside a receptive ovule (Ge *et al.*, 2017). Pollen tube growth requires rapid controlled and organized deposition of different cell wall materials while maintaining a balance between turgor and cell wall resistance (Winship *et al.*, 2011; Chebli *et al.*, 2012). Because of the extreme speed of the growth process, this balance is easily disturbed, resulting in bursting. Any bursting prior to reaching a female gametophyte leads to the loss of the sperm cells and failure to fertilize. Pollen tube integrity is thus a nominal score obtained by determining whether a pollen tube tip is intact or if it has burst precociously.

#### Ability to reach ovule, sperm cell release and seed set.

While *in vitro* pollen germination indicates the ability of a pollen grain to germinate and produce a pollen tube, it does not assess the ability of a pollen tube to navigate towards a female gametophyte and release the sperm cells. Targeted growth and sperm cell delivery require several cellular functions – the ability to perceive and respond to directional guidance cues and the ability to burst once the female gametophyte is reached (Higashiyama and Hamamura, 2008). Many mutants exist in which these functions are impaired, although pollen tube elongation occurs (Kessler and Grossniklaus, 2011). It is therefore reasonable to assume the heat stress administered during any phase of pollen development has the potential to interfere specifically with this final step of the progamic phase. *In vivo*, the ability to target the female gametophyte and release sperm cells can be assessed by scoring seed set or by observing pollen tube bursting vs overgrowth events (Leydon *et al.*, 2015). Semi-*in vivo* assays can be employed for this purpose as well and can be used to investigate the function of genes and proteins of interest involved in micropylar guidance. For ovule targeting assays, mature ovules are placed in front of the cut end of the excised stigma–style. Pollen tubes receive directional cues from their respective target ovules, enabling species-specific attraction (Higashiyama and Yang, 2017). Using live cell imaging, ovule targeting permits observation of pollen tube reception, synergid degradation and semi-*in vivo* gamete fusion. In comparison, pollen tube attraction assays directly determine the capacity of a specific molecule to attract or repulse pollen tubes. Detailed protocols for both approaches have been outlined by Zhong *et al.* (2020).

### Pollen vigour

Pollen vigour has been described as the time taken for germination and the time necessary for the tube to reach the embryo sac (Shivanna *et al.*, 1991*a*). Pollen vigour is an ordinal measure and is usually time-dependent, either in terms of absolute time (e.g. time for pollen to germinate) or relative velocity with regard to competing pollen tubes. In mixed pollinations and when the number of pollen grains on a stigma exceeds that of the receptive ovules in an ovary, the time required to germinate, grow a tube and reach an ovule to complete double fertilization is critical in determining whether the genetic information of a given pollen grain will be passed on to the next generation. Larger pollen loads can intensify selection among gametophytes such that more intensive pollen competition will produce more vigorous progeny (Zhang *et al.*, 2010). In tomato (*Solanum lycopersicum*) and pepper (*Capsicum annuum*), a correlation between viability and pollen tube length was found, suggesting that pollen batches of higher quality tend to contain more vigorous pollen tubes, forming longer pollen tubes (Langedijk *et al.*, 2023). Pollen vigour can be tested through *in vitro*, semi-*in vivo* and *in vivo* pollen germination assays by measuring the length of a pollen tube grown in a given time interval or by measuring how much time it takes for pollen to germinate and/or produce a tube of a certain length. Furthermore, pollen vigour can be examined through scoring pollen tube morphology, which can be reflected in the degree of uniformity of pollen tube diameter, the structure of the cell wall, or the directionality of tube growth.

#### In vitro and semi-in vivo pollen germination.

 Semi-*in vivo* germination is an experimental approach that exploits the style–stigma interaction to capacitate the pollen (Sankaranarayanan and Higashiyama, 2018; Johnson *et al.*, 2019) and to then let the tubes grow into a transparent *in vitro* medium for observation of pollen tube growth and scoring pollen tube length. This is done by dissecting the pistil of an unpollinated or sterile plant, hand-pollinating it, and placing it onto a germination medium to permit pollen tubes to grow through the stigma and transmitting tissue and into the medium. Semi-*in vivo* and *in vitro* pollen germination experiments can be performed to determine either instantaneous or average/overall pollen tube growth rates. Instantaneous pollen tube growth rate requires recording the dynamic changes in elongation rate of individual tubes, which is a time-consuming approach. Average or overall pollen germination can be measured by dividing the length of a pollen tube by the time it takes to reach that length. It is important to note that in this case, time 0 should be the moment of germination, not pollination or imbibition. Pollen vigour can also be expressed as the time the pollen requires to germinate from the moment it is hydrated, as was done in cherimoya (*Annona cherimola*). Germination time in this species was found to strongly depend on the interval that had elapsed since dehiscence (Rosell *et al.*, 2006).

Semi-*in vivo* experiments can also be used to investigate the efficiency of pollen tubes to target ovules if these are placed into the growth medium at distances that allow diffusion of signalling components to the elongating tubes (Zhong *et al.*, 2020). Consequently, semi-*in vivo* trials can be used to investigate how high temperatures administered during the progamic phase affect pollen germination and tube growth. Semi-*in vivo* germination experiments performed on stressed *Nicotiana tabacum* pollen showed pollen viability not to be affected by exposure to high relative humidity (>95%) and high temperature stress (38 or 45 °C), but pollen vigour to be significantly affected (Shivanna *et al.*, 1991*b*). At both 38 and 45 °C, pollen germinated on the stigma for semi-*in vivo* assays, but the pollen tubes took much longer when compared with the controls (21 °C) to reach the ovary. Moreover, in *in vitro* germination tests of the same species, pollen treated at 38 °C took longer to germinate than at control conditions (21 °C) (reduced vigour), while pollen grains treated at 45 °C failed to germinate i*n vitro* (reduced viability). This confirms that *in vitro* germination conditions make for less robust pollen behaviour compared with germination on the stigma. In another study, cotton plants were exposed to high temperature stress (40/34 °C day/night) for 3 d at different developmental stages to determine relative susceptibility to heat of the different stages of male gametophyte development (Song *et al.*, 2015). After heat treatment, plants were returned to control growing conditions and pollen grains were collected for 40 consecutive days to determine viability through *in vitro* germination assays. Temperatures above 35 °C during microsporogenesis were found to influence developmental stages from the sporogenous cell to tetrad, as well as strongly inhibit pollen tube growth in the style.

#### Pollen tube morphology.

Pollen tube morphology and growth behaviour can reveal insights into pollen vigour by predicting the ability to efficiently and effectively grow towards its target. Pollen tube morphology can be observed by performing *in vitro*, *semi-in vivo* and *in vivo* pollen experiments. A study examining how temperature stress impacts pollen tube integrity in wild-type tomato and the *are* mutant (reduced flavonol accumulation in pollen grains and tubes) detected morphological changes in mutant pollen tubes observed as swelling at the tip and impaired integrity of the tube (reduced vigour), frequently resulting in rupture (reduced viability) (Muhlemann *et al.*, 2018).

Pollen tube morphology can also be scored by visualizing structural cell components. Tobacco pollen tube growth is less sensitive to heat stress than germination (Parrotta *et al.*, 2016), but damage is observed in microtubules and actin filaments, affecting particularly the subapical actin array, a region critical in determining organelle and vesicle content in the pollen tube apex and consequently cell wall assembly (Chebli *et al.*, 2012; Cameron and Geitmann, 2018). As a result, the typical apical–distal gradient in the degree of pectin esterification is lost, and pectin (detected by PI label) appears to excessively accumulate. In *Echinopsis chamaecereus* (Cactaceae), pollen tubes exposed to heat stress (35 °C or 40 °C) exhibited enhanced callose accumulation in the pollen tube tip when compared with the control (Çetinbaş-Genç, 2020). Additionally, at 40 °C, actin filaments exhibited a change in spatial distribution, losing their parallel organization and presenting disorganizations in the apex.

Pollen tube morphology encompasses a qualitative approach to scoring samples, through descriptive reporting of pollen tube growth behaviour and patterns. To disentangle the effect of stress on pollen tube growth behaviour from that on germination, pollen tube morphology should be assessed once a pollen tube is well established (typically when it has achieved a length greater than the diameter of the pollen grain). Aberrant morphological features can distinguish between phenomena specific to the apex, the subapical region or the shank region of the elongating tube. Healthy pollen tubes usually exhibit straight growth with a tube uniform in diameter. Stressed pollen tubes may show swelling in any region of the pollen tube indicative of suboptimal regulation of the tip growth process (Muhlemann *et al.*, 2018). Stressed pollen tubes may also exhibit a zigzagging or meandering behaviour, ultimately resulting in a slower approach of their target, although this behaviour has also been interpreted to serve to detect gradients of signalling molecules in the growth substrate (Cameron and Geitmann, 2018). Quantification of pollen tube morphology is possible for certain features (e.g. fluorescence intensity and spatial distribution of label for pollen tube cell wall components) (Çetinbaş-Genç, 2020). More detailed investigations into how heat stress affects cell wall assembly and composition will help identify genes regulating the ultra-rapid and efficient tip growth process in this cell.

#### Competition experiments.

Competition among pollen with different genotype in reaching ovules and achieving fertilization reveals relative fitness. This is particularly relevant when the pollen load on a stigma is high and only the fastest-growing pollen tubes achieve fertilization (Mulcahy, 1979). A simple competition experiment can be performed using heterozygous pollen by quantifying transmission to the next generation. Alternatively, mixed batches of pollen grains from different lines can be prepared, although the exactitude of relative quantities of pollen applied to the stigma is likely an important source of experimental variation. In cherry (*Prunus avium*), high temperatures (30 °C) were shown to affect pollen tube dynamics and consequently influence the proportion of pollen tubes to successfully reach the ovary (Hedhly *et al.*, 2004). Pollen competition is affected by high temperatures through differential impact on pollen adhesion, germination and tube growth. The severity of damage is influenced by the male genotype, female recipient and temperature–male and temperature–female genotype interactions (Hedhly *et al.*, 2005; Selak *et al.*, 2013; Montalt *et al.*, 2019). Due to the different susceptibility of individual genotypes to high temperatures, heat stress acts as a selective pressure for genotypes with better pollen performance which can be useful for breeding programmes focused on improved adaptation to different temperature conditions.

## Prediction models: response to temperature and analysis

Predictive modelling can be used to interpret incomplete datasets and estimate unknown parameters (Table 2). Regression analysis and cardinal temperatures can be used to describe an organism’s response to a range of temperatures, even if not all relevant temperatures are tested. This is achieved by fitting different models (i.e. linear, second-degree polynomial) to available data to predict the optimal temperature, the cold temperature that prevents germination at the lower end of the range, and the lethal high temperature.

**Table 2.** Analysis of pollen performance under various temperature treatments.

Method of analysis	Methods of measuring
Prediction models: response to temperature and analysis	Cardinal temperatures (T_max_, T_min_, T_opt_): Kakani *et al.*, 2002, 2005; Liu *et al.*, 2006; Reddy and Kakani, 2007; Salem *et al.*, 2007; Acar and Kakani, 2010; Hebbar *et al.*, 2018; Ranasinghe *et al.*, 2018 Cumulative stress response index (CSRI): Kakani *et al.*, 2005; Koti *et al.*, 2005; Salem *et al.*, 2007; Reddy and Kakani, 2007; Çetinbaş-Genç, 2020 Principal component analysis (PCA): Kakani *et al.*, 2002, 2005; Jiang *et al.*, 2015; Ranasinghe *et al.*, 2018

The cumulative stress response index (CSRI) and principal component analysis (PCA) are statistical approaches to interpreting data/parameters of an experiment to help reveal which populations are most impacted by the stressor. Using CSRI or PCA, pollen fitness for different genotypes of a species can be determined, enabling sorting or classification of the genotypes into groups (i.e. heat sensitive, heat tolerant).

### Cardinal temperatures

Cardinal temperatures describe the range in which a particular species can perform a particular biological process, such as the capability of pollen to germination or facilitate pollen tube growth, by defining maximum, minimum, and optimum temperatures (T_max_, T_min_, T_opt_). Values for a range of temperatures are established experimentally and are then analysed using different linear and non-linear regression models to determine best fit. The effects of temperature on pollen germination and pollen tube length have been analysed using linear and non-linear regression models, which are commonly used to quantify developmental responses of biological organisms to temperature (Koti *et al.*, 2005). The best fit is often characterized by the greatest *R*^2^ value and smallest root mean square deviation (RMSD) for observed and fitted values. The quadratic model and modified bilinear model best describe pollen developmental response to temperature and can be used to estimate the parameters of cardinal temperatures. T_min_ and T_max_ are characterized as lethal minimum temperature and lethal maximum temperature, at which no pollen performance occurs, corresponding to the *x*-intercepts for the fitted model. T_opt_ is found at the maximum point of the graph (vertex, peak), depicting the temperature at which optimal pollen performance occurs. Across multiple studies analysing cultivars of coconut (Hebbar *et al.*, 2018), *Capsicum* (Reddy and Kakani, 2007), cotton (Kakani *et al.*, 2005; Liu *et al.*, 2006), groundnut (Kakani *et al.*, 2002) and *Pistacia* spp. (Acar and Kakani, 2010), the modified bilinear equation was determined to best fit the data (i.e. generated the highest *R*^2^ value and smallest RMSD) for both pollen germination and pollen tube length. However, in soybean linear and non-linear regression models were analysed for maximum pollen germination and pollen tube lengths and to determine the best fit to the quadric and modified bilinear model (Salem *et al.*, 2007).

### Cumulative stress response index

The CSRI is defined as the sum of relative individual component responses under each treatment and evaluates the response of an organism to a treatment in comparison to a control treatment (Koti *et al.*, 2005). The cumulative temperature response index (CTRI) is more specific and describes the response of an organism to temperature treatments against a control temperature. In the context of pollen performance, this technique uses all pollen parameters of interest to identify species variability to high temperatures. The scores retrieved from this calculation can be used to evaluate and classify the different genotypes used in a study. For example, a study examining the effect of heat stress on pollen may investigate parameters such as maximum pollen germination rate, maximum pollen tube length and mean cardinal temperatures (T_min_, T_opt_, T_max_) of pollen germination and pollen tube length, which can be input into a formula for CSRI (eqn 1). The results from germination experiments can be used to calculate the cumulative temperature response index of each genotype by finding the difference between the temperature and control, divided by the control. The CSRI values generated for each of the cultivars can be ranked on a scale. A lower CSRI score describes a larger difference between the treatment and control conditions, and a higher score indicates a smaller difference between the treatment and control conditions. Therefore, genotypes with a low CSRI score would be considered more heat-sensitive than genotypes with a high CSRI score, which would be heat-tolerant. Genotypes are classified based on the sum of CSRI over all treatments (Total Stress Response Index, TSRI) as either tolerant (> minimum TSRI − 1 standard deviation), intermediate (> minimum TSRI − 2 standard deviations and < minimum TSRI − 1 standard deviation) or sensitive (< minimum TSRI − 2 standard deviations) (Koti *et al.*, 2005).


$$\begin{array}{lll} CSRI= & [\frac{PG_{t}}{PG_{h}}+\frac{PTL_{t}}{PTL_{h}}+\frac{PG_{opt_{t}}}{PG_{opt_{h}}}+\frac{PG_{max_{t}}}{PG_{max_{h}}}+\frac{PG_{min_{t}}}{PG_{min_{h}}}\; & +\frac{PTL_{opt_{t}}}{PTL_{opt_{h}}}+\frac{PTL_{max_{t}}}{PTL_{max_{h}}}+\frac{PTL_{min_{t}}}{PTL_{min_{h}}}] \end{array}$$ (1)

Equation (1) is an example of a formula used to calculate the CSRI, where PG is pollen germination percentage; PTL is pollen tube length; opt, max and min indicate the cardinal temperatures at which PG and PTL were measured; t indicates the genotype; and h indicates the maximum value for PG or PTL observed over all genotypes.

In soybean, 44 genotypes were analysed and grouped according to their heat tolerance using CTRI, calculated as the sum of eight individual stress responses (ISRs) derived from maximum pollen germination and maximum pollen tube length after a 24-h incubation time, T_max_, T_min_ and T_opt_ for pollen germination and pollen tube length (Salem *et al.*, 2007). Soybean genotypes with lower CTRI values (6.76–7.11) were determined as sensitive to heat (as a result of their lower ability or smaller impact on whole response), while genotypes with higher CTRI values (7.50–7.31) were established to be tolerant to heat, and genotypes with CTRI values in between (7.12–7.30) were determined to be intermediate. In a separate study, screening *Capsicum* species for high temperature tolerance, only one genotype was found to be tolerant to high temperatures (CTRI > 7.52) while two genotypes exhibited an intermediate response (CTRI = 7.36–7.52) and four genotypes displayed sensitivity to high temperatures (CTRI = 7.18–7.35) (Reddy and Kakani, 2007).

### Principal component analysis

PCA is a statistical method used for analysing large datasets containing multiple correlated variables by reducing the dimensionality of the datasets while increasing their interpretability, but at the same time minimizing information loss (Jolliffe and Cadima, 2016). By reducing a large dataset, PCA can be useful when trying to run an algorithm through the data and/or visualize a complex dataset. PCA can use parameters of pollen germination and pollen tube length [e.g. cardinal temperatures (T_min_, T_opt_ and T_max_)] to identify which parameters best describe tolerance to temperature. Using PCA, classification of seven coconut hybrids was performed on the basis of their temperature tolerances and divided into three groups: tolerant, moderately tolerant, and less tolerant for heat stress (Ranasinghe *et al.*, 2018). Out of eight pollen germination and pollen tube growth parameters, PCA identified T_max_ for pollen germination and T_opt_ for pollen tube growth as the most important pollen parameters describing varietal tolerance to high temperature in coconut hybrids (performed by PRINCOM procedure in SAS 9.1 software). In groundnut (*Arachis hypogaea*), 21 genotypes were subject to *in vitro* germination assays in order to identify differences in pollen tolerance to temperature (Kakani *et al.*, 2002). PCA classified genotypes as either heat-tolerant or heat-susceptible and identified maximum percentage pollen germination and pollen tube length of the genotypes, and T_max_ for the two processes as the most important pollen parameters in describing genotypic tolerance to high temperatures. PCA has been used to identify heat tolerant/heat susceptible genotypes in other species, including cotton (Kakani *et al.*, 2005) and pea (*Pisum sativum*) (Jiang *et al.*, 2015).

## Confounding factors and experimental design

The ability of pollen grains to germinate depends on the genotype, the conditions during plant and pollen development, and the conditions under which the pollen germinates. Plant growth and development are different for plants grown in a field setting compared with plants grown in a temperature/humidity/carbon dioxide-controlled chamber. In the field, high temperatures are often accompanied by other abiotic stressors, such as drought stress or high light intensities (Raja *et al.*, 2019). In tobacco, plants were exposed either to a single stress, heat (37 °C for 1 h followed by an increase to 44 °C for 6 h) or drought (withdrawing water from plants until they reached a relative water content of 65–70 %; typically 6–7 d), or to both stressors simultaneously (Rizhsky *et al.*, 2002). Transcriptomics demonstrated that the response of plants to a combination of heat stress and drought is different from the responses of plants to each stressor applied individually. This means that disentangling genetic from abiotic effects on pollen viability is particularly important if the pollen-producing plants are grown in the field. Furthermore, careful attention must be taken when designing experiments investigating stage-specific thermosensitivity of the male gametophyte, especially when short-term heat stress is applied.

Thermotolerance, the ability of a plant to preserve its physiological status under heat stress, may occur through priming, in which the plant is exposed to sub-lethal temperatures or a progressive increase in temperature (Mesihovic *et al.*, 2016). Whether heat stress causes a positive or negative effect depends on the thermic threshold of the organism, its state of acclimation and adaptation, and the severity and duration of stress – typically with heat stress occurring when temperatures rise 5–15 °C above the optimum for plant growth and development (Kranner *et al.*, 2010; Fragkostefanakis *et al.*, 2016). Based on the magnitude and duration of applied stress, plants can activate transient or long-term response mechanisms to induce protection and recovery from the stress. Acquired thermotolerance can be triggered through a pre-stress treatment that activates a heat stress response involving the activation of different heat shock factors that in turn facilitate the synthesis of heat-responsive genes. This can ultimately positively affect pollen performance under heat stress by altering and preparing its metabolism to cope with the stress. When designing a study, it is essential to determine an appropriate heat stress regime (e.g. heat shocks, heat waves, long-term warming scenarios, ramping of temperatures over time), the method of application, and at which stage of development the stress is applied. Short-duration heat shock treatments (hours or shorter) are the most common form of heat stress used on both cultivated and wild species. They induce the heat shock response, including rapid synthesis of heat shock proteins and molecular chaperones (Jagadish *et al.*, 2021). Adverse environmental conditions affect all stages of male gametophyte development and, given that pollen development occurs inside the anther, stress administered to the sporophyte can also indirectly affect the gametophytes (Mesihovic *et al.*, 2016; Hinojosa *et al.*, 2019). Critically for meta-analyses, pooling and interpreting data from samples that received heat stress at different developmental stages and/or under different temperature regimes may generate misleading conclusions. Consequently, the administration protocol of a heat stress treatment during pollen development should define and report during which developmental stage exactly it is applied: (1) pollen mother cells to tetrads, (2) early microspores to early bicellular pollen, (3) late bicellular/tricellular (mature) pollen, and (4) germinating pollen (Mesihovic *et al.*, 2016).

Pollen vigour is impacted by environmental conditions experienced during storage, including temperature, humidity, oxygen, light, and the nature of the storage container. Furthermore, the duration of storage (or age since dehiscence) is a crucial factor. Pollen grains of *N*. *tabacum*, *Agave* sp., *Tradescantia virginiana* and *Iris* sp. stored under laboratory conditions (21 ± 2 °C) were tested for FCR and *in vitro* germination at 10-d intervals to determine the effect of pollen storage time on germination rate (Shivanna *et al.*, 1991*a*). Fresh pollen showed >90 % FCR and >80 % germination within 1 h, and pollen grains stored for 10 d did not show any decline in FCR or in the speed of germination. However, pollen samples stored for 20 d, while not showing a decline in FCR value or germination rate, took much longer to germinate. One hour after imbibition, only ~6 % of the stored pollen had germinated compared with a near-100 % rate for the fresh pollen grains. Maximum germination in the stored pollen sample was reached only by 6 h.

Experiments using cellular proxies as determinants of pollen viability (e.g. FCR test, Alexander’s stain) have considerable limitations in their capacity to accurately predict the ability of pollen to germinate. Commonly cited is the issue arising with immature pollen, which tends to produce false-positive results, but this effect varies with the species. Consequently, selection of a method to test pollen performance and optimization of experimental protocols are important when considering what questions are being asked, and thus what data should be collected (Table 3). For example, a pollen grain may be viable in principle but unfavourable conditions in the *in vitro* test assay (e.g. sub-optimal germination media; rehydration period too short) prevent this from happening. Unless a protocol is optimized for a given species, it might result in a low overall germination rate that makes it difficult to detect differences between temperature-treated and control samples. Staining pollen grains is an effective approach to estimate pollen viability; however, due to the potential of false-positives (staining non-viable pollen grains as viable), it may be worthwhile using multiple dyes or to pair staining tests with *in vitro* germination experiments. Another point to consider is that while staining pollen samples is fast and easier to conduct than *in vitro* growth assays, determining viability rate based on a proxy may not reveal enough information to respond to the needs of a given research project. In these cases, *in vitro* germination assays are necessary to observe the actual viability of pollen, including pollen germination and pollen tube growth. Overall, *in vitro* pollen germination experiments are a more reliable indicator than stain-based tests as the latter only determine whether the pollen contains the necessary enzymes or cell structures needed to initiate germination, while germination tests determine the actual capacity to form a pollen tube.

**Table 3.** Advantages and disadvantages of various tests to assess pollen performance through detection of cellular proxies or *in vitro* germination.

Test	Advantages	Disadvantages	Considerations
Detection of cellular proxies	Fast and simple High sensitivity; generates images with high contrast	Short lifetime of some dyes: FDA working solution appears to lose its properties as soon as 1 h after preparation (Pinillos and Cuevas, 2008). Varying degrees of fluorescence signal intensity, difficulty in recognizing some of the non-fluorescing grains that appear almost as dark as the background; underscoring non-fluorescing grains tends to overestimate the fluorescing grains in the sample unless brightfield micrographs are used for pollen count (Sukhvibul and Considine, 1993; Khatun and Flowers, 1995) May generate false positives (i.e. stain dead pollen grains) (Käpylä, 1991). Microscopy-related artefacts (e.g. photobleaching, signal bleed-through, phototoxicity).	Pollen quality: a high correlation between a staining test and pollen germination rate can only be expected when mature pollen is used; immature pollen yields false positives (Heslop-Harrison *et al.*, 1984). Choice of fluorophore for staining; staining protocol. Available instruments for imaging.
*In vitro* germination	Rapid, simple (computer automation speeds up analyses). Commonly used technique, many protocols optimized and available (e.g. germination media protocols; scoring protocols). In many species this parameter shows correlation with fruit set and seed set (Visser, 1955).	Susceptible to environmental conditions in the experimental setup (temperature, relative humidity). Pollen stored in unfavourable conditions may have lower germination rate, delay in germination (Shivanna *et al.*, 1991*a*). False negatives: certain pollen may not germinate *in vitro* (if *in vitro* procedures are not optimized) although the pollen is viable.	Optimization of germination medium required for each species. Absence of biological features involved in *in vivo* double fertilization (i.e. microstructure and chemistry of transmitting tract, signalling molecules emitted by female sporophytic tissues and female gametophyte).

After optimizing germination media, temperature experiments on pollen using *in vitro* germination can be organized in a variety of ways to change different parameters of a temperature treatment. Some considerations include determining what temperature will be used to incubate control samples (e.g. room temperature, 25 °C), how long it takes for the pollen to germinate *in vitro* under control vs high-temperature conditions (i.e. a delay in pollen germination due to unfavourable conditions), and when the high temperature stress is applied to pollen samples (e.g. during pollen development and/or the progamic phase). Lastly, any external factors (e.g. temperature fluctuations, changes in humidity, pests) should be reviewed to determine if pollen development may have been affected outside of experimental parameters.

## CURRENT AND FUTURE PERSPECTIVES

Due to climate change, overall global temperatures are expected to increase and incidences of heat waves to become more frequent, threatening crop productivity and food security. High temperatures affect plant reproduction and directly impact fruit set (Dane *et al.*, 1991). Therefore, the adverse impacts on plant reproduction due to high temperature stress have global implications on crop production systems as well as implications for the geographic distribution of natural plant populations. Here we have outlined different methods to screen for thermotolerance in pollen that can be used to determine heat-tolerant and heat-susceptible genotypes, and to detect and predict reproduction-related impacts of climate change on agricultural and wild plant performance. Standardizing scoring methods for pollen performance and discerning the advantages and disadvantages of each technique enable replicable, reliable and reproducible experiments for both *in vitro* and *in vivo* studies. Understanding the various biochemical and genetic factors governing the heat stress response in pollen is an important step in developing effective breeding strategies to permit optimal yields and stable production across various crop species. More specifically, different approaches, such as genomics, transcriptomics, proteomics and metabolomics, can take screening further to enable comprehensive detection of genes, transcripts, proteins and metabolites involved in the heat stress response. These methods are complementary and can be used in combination to create a system-level overview of different genotypes more tolerant to high temperature stress. Using a multi-omics approach, including lipidomic, metabolomic and transcriptomic analysis, it was observed that the heat stress response in pollen is a complex and multi-layered trait requiring interactions of different cellular processes, highlighting, for example, that rapid lipid remodelling not controlled transcriptionally is equally important as metabolomic adaptations and transcriptional changes (Krawczyk *et al.*, 2022). Through identification of heat-tolerant pollen genotypes, breeders and farmers can select suitable varieties that can be cultivated in different regions faced with specific abiotic stressors.

With regard to natural populations, an increased understanding of the reproductive process under heat stress will enable improved prediction of the effects of climate change on population dynamics (Gillespie *et al.*, 2012), geographical distribution (Castellanos-Frías *et al.*, 2016), potential success and spread of invasive plants (French *et al.*, 2017) and pollinator–plant interactions (Walters *et al.*, 2022).

Plant pollination and fertilization are complex, multi-step processes that are controlled at several checkpoints. When these processes are subject to unfavourable environmental conditions, there may be a reduction in available resources (e.g. water for pollen rehydration and pollen tube growth) that influence the success of pollen grains, ultimately increasing competition for reproductive success (Chaturvedi *et al.*, 2021). While performing heat stress experiments *in vitro* can provide important insight into the various mechanisms governing heat stress response, it is important to acknowledge and identify the differences in response of plants grown in natural settings (e.g. field), as opposed to plants grown in controlled conditions (e.g. greenhouse, growth chamber). Often, multiple abiotic stressors coincide and experiments solely looking at heat stress may be limited in their conclusions.

Future work should investigate the pathways involved in heat stress response *in vivo*, which can be achieved by performing semi-*in vitro* assays investigating pollen fitness and stress responses at gene regulatory and metabolic levels, such as transcriptome profiling and proteomic and metabolic analysis between heat-sensitive and heat-tolerant plants. The heat shock response and particularly the epigenetic regulators (transcription factors) and signalling pathways involved remain poorly understood. Future studies should design experiments that best mimic real-life environmental conditions so the resulting data can be implemented by breeders to select crops best suited to growing in a given region. This includes developing experiments with co-occurring stresses (e.g. abiotic and biotic stress) to yield results similar to field conditions breeders can use to select crops suited to tolerate these conditions. By using different statistical methods, such as CSRI and PCA, identification of heat tolerant vs heat susceptible crops can be achieved and these genotypes can be isolated to be further examined. Identification of specific genotypes tolerant to high temperature stress can be used by breeding programmes to grow crops that cope better with climate change.
